# Nodule and Root Zone Microbiota of Salt-Tolerant Wild Soybean in Coastal Sand and Saline-Alkali Soil

**DOI:** 10.3389/fmicb.2020.523142

**Published:** 2020-09-22

**Authors:** Yingjie Yang, Lei Liu, Raghvendra Pratap Singh, Chen Meng, Siqi Ma, Changliang Jing, Yiqiang Li, Chengsheng Zhang

**Affiliations:** ^1^Marine Agriculture Research Center, Tobacco Research Institute, Chinese Academy of Agricultural Sciences, Qingdao, China; ^2^Bureau of Agriculture and Rural Affairs of Laoshan District, Qingdao, China; ^3^Department of Research and Development, Biotechnology, Uttaranchal University, Dehradun, India

**Keywords:** wild soybean, salinity, root nodule, microbiome, illumina sequencing, 16S rRNA, *nifH*

## Abstract

Soil salinization limits crop growth and yield in agro-ecosystems worldwide by reducing soil health and altering the structure of microbial communities. Salt-tolerant plant growth-promoting rhizobacteria (PGPR) alleviate plant salinity stress. Wild soybean (*Glycine soja* Sieb. and Zucc.) is unique in agricultural ecosystems owing to its ability to grow in saline-alkali soils and fix atmospheric nitrogen *via* symbiotic interactions with diverse soil microbes. However, this rhizosphere microbiome and the nodule endosymbionts have not been investigated to identify PGPR. In this study, we investigated the structural and functional rhizosphere microbial communities in saline-alkali soil from the Yellow River Delta and coastal soil in China, as well as wild soybean root nodule endosymbionts. To reveal the composition of the microbial ecosystem, we performed 16S rRNA and *nifH* gene amplicon sequencing on root nodules and root zones under different environmental conditions. In addition, we used culture-independent methods to examine the root bacterial microbiome of wild soybean. For functional characterization of individual members of the microbiome and their impact on plant growth, we inoculated isolates from the root microbiome with wild soybean and observed nodulation. *Sinorhizobium/Ensifer* accounted for 97% of the root nodule microbiome, with other enriched members belonging to the phyla Actinobacteria, Bacteroidetes, Chloroflexi, Acidobacteria, and Gemmatimonadetes; the genera *Sphingomonas*, *Microbacterium*, *Arthrobacter*, *Nocardioides*, *Streptomyces*, *Flavobacterium*, *Flavisolibacter*, and *Pseudomonas*; and the family Enterobacteriaceae. Compared to saline-alkali soil from the Yellow River Delta, coastal soil was highly enriched for soybean nodules and displayed significant differences in the abundance and diversity of β-proteobacteria, δ-proteobacteria, Actinobacteria, and Bacteroidetes. Overall, the wild soybean root nodule microbiome was dominated by nutrient-providing *Sinorhizobium/Ensifer* and was enriched for bacterial genera that may provide salt resistance. Thus, this reductionist experimental approach provides an avenue for future systematic and functional studies of the plant root microbiome.

## Introduction

Soil salinization is a common abiotic stress that restricts crop growth and yield in agro-ecosystems worldwide. The Yellow River Delta is one of the three largest river deltas in China and is becoming a major region for agricultural development (Jing et al., 2019); however, crop production is limited by high soil salinity, which reduces the water-extraction capacity of roots and has a devastating effect on plant metabolism. In addition, high soil salinity disrupts cellular homeostasis and results in the uncoupling of major physiological and biochemical processes; thus, the reclamation of stressed soils is critical for meeting the food demands of the ever-increasing population and improving soil quality (Li et al., 2016b; Etesami and Maheshwari, 2018).

The microbiome that symbiotically inhabits the interior of plant roots and saprophytically interacts with soil particles in the rhizosphere is vital for promoting plant growth, fixing nitrogen *via* nodulation, and protecting plants from stress. Salt-tolerant plant growth-promoting rhizobacteria (PGPR) have displayed great potential for alleviating plant salinity stress (Akram et al., 2016; Backer et al., 2018; Chu et al., 2019; Kearl et al., 2019; Rodriguez et al., 2019). These beneficial soil microbes reside in the rhizosphere and, together with root exudates, can provide plants with nutrients, growth hormones, antioxidants, and systemic resistance, even under high salt concentrations (Ahmad et al., 2013; Li et al., 2014; Kong et al., 2015; Agler et al., 2016). Indeed, salt-tolerant PGPR and their metabolites isolated from halophyte species in saline soils can play key roles in mitigating salinity stress and enhancing crop yield (Bouhmouch et al., 2005; Albdaiwi et al., 2019; Chauhan et al., 2019; Chen et al., 2019; Saghafi et al., 2019). Different microbes have been associated with various plants and growth environments, suggesting the existence of specific microbe-host interactions (Perez-Jaramillo et al., 2019). In addition, some endophytes, including *Sphingomonas*, *Bacillus*, *Enterobacter*, and *Pantoea* species, can stimulate plant growth under saline conditions (Li et al., 2016a). Moreover, diazotrophs have been isolated from the nodule and root surface of legumes or the root surface of other plants (Fan et al., 2018). The same plant is generally associated with more than one diazotroph, which display different patterns of regional distribution and frequency. The most common diazotrophs identified so far are *Azospirillum*, *Herbaspirillum*, *Enterobacter*, *Klebsiella*, *Azotobacter*, *Beijerinckia*, *Bacillus*, and *Pseudomonas*, while other diazotrophs belong to the *Lactobacillus* and *Halobacillus* (Dos Santos et al., 2012).

Wild soybean (*Glycine soja* Sieb. and Zucc.) is widely distributed throughout China, northeast Russia, Korea, and Japan (Ge et al., 2010; Jing et al., 2018; Xie et al., 2019) and is characterized by greater cold hardiness, salt tolerance, and disease resistance than cultivated soybean *Glycine max* Merr (Jing et al., 2018; Xie et al., 2019). Consequently, wild soybean is of high economic value, particularly for the cultivation of advanced soybean varieties (Jing et al., 2018; Ohigashi et al., 2019); however, its root zone microbiome and nodule endosymbionts have not yet been investigated to identify PGPR by next generation sequencing.

Nitrogenases are widespread in bacteria and archaea, providing them with a competitive advantage in environments depleted of bio-available nitrogen, which affects PGPR function (Dos Santos et al., 2012; Fan et al., 2019). The ability to fix nitrogen is widely, but sporadically, distributed among archaea and bacteria, including the families Proteobacteria, Firmicutes, Cyanobacteria, Actinobacteria, and Chlorobi (Dos Santos et al., 2012). *NifH* is used as a marker gene to detect nitrogen-fixing microorganisms in the environment (Igai et al., 2016; Fan et al., 2019), and nitrogen-fixing Rhizobiales have been identified in the special root nodules of crop legumes, such as alfalfa, beans, peas, and soy, which provide 20% of food protein worldwide (Mnasri et al., 2012; Egamberdieva et al., 2013; de Almeida Lopes et al., 2016; Sharma et al., 2016; Singh et al., 2019).

In this study, we combined metagenomic approaches and *NifH* Illumina sequencing to characterize wild soybean rhizosphere and nodule microbiomes at a deep taxonomic resolution. In addition, we investigated the structure and function of the microbiota at the wild soybean root-soil interface and in the nodules of coastal sand and saline-alkali soil using comparative computational approaches. Metagenomic results were correlated and confirmed by the culture and isolation of microbial communities, while their effect on nodulation was verified using root infection assays. Together, this reductionist experimental approach provides an avenue for future systematic and functional studies of the plant root microbiome.

## Materials and Methods

### Sampling Sites

The samples used in this study were obtained from the Yellow River Delta (37°39′43.58″N, 118°40′48.19″E, north of the Shangdong Peninsula, China) and coastal sand from Qingdao near the Huanghai sea coastline (36°7′58″N; 120°26′42″E, south of the Shangdong Peninsula, China). Soil salinity, electrical conductivity (EC), and pH were measured using a 1:2.5 soil:water solution. Briefly, 10 g of air-dried soil was dissolved in 25 ml of ddH_2_O, mixed completely for 30 min, and filtered using filter paper. The filtrate was measured using pH and conductivity meters before being evaporated to obtain salt. According to the Food and Agriculture Organization (FAO of the United Nations) World Reference Base for Soil Resources, the soil from the Yellow River Delta was calcaric fluvisol and had a pH of 7.94, soil salinity of 1.96, soil organic matter of 1.01%, and total nitrogen of 0.11/100 g (Jing et al., 2019), with an EC of 565 ± 33 μS/cm. The coastal sand had a pH of 6.5, soil salinity of 0.56%, soil organic matter of 0.41%, total nitrogen of 0.60 mg/100 g, and EC of 155 ± 64 μS/cm. Although these soils were not highly salinized, cultivated soybean was not able to grow normally, yet wild soybean was able to grow normally and produce yield. Since rhizospheric soil was not readily obtained from the sand samples, we used the root zone for further experiments. Briefly, surface soil (2 cm deep) was removed and soil within 5 cm of the plant stem was moved using a shovel. The root was then lifted out carefully, with surrounding soil designated as the root zone. Root-attached nodules were washed thoroughly with sterilized distilled water and 50 nodules were sent for sequencing.

### Library Preparation, Sequencing, and Bioinformatic Analysis of High-Throughput Data for the Rhizospheric Soil and Nodules

Total metagenomic DNA was extracted from rhizospheric soil and nodule samples using a FastDNA spin kit for soil (MP Biomedicals, LLC, Santa Ana, CA, USA) according to the manufacturer’s protocols and verified using 0.8% agarose gel electrophoresis. Extracted DNA was amplified using a 799F (5'-AACMGGATTAGATACCCKG-3') and 1193R (5'-ACGTCATCCCCACCTTCC-3') universal primer set targeting the V5–V7 region of the bacterial 16S ribosomal RNA (rRNA) genes as well as a *nifH*1 460–476 (5'-ADNGCCATCATYTCNCC-3') and *nifH*2 115–131 (5'-TGYGAYCCNAARGCNGA-3') universal primer set targeting the *nifH* genes (Zehr and McReynolds, 1989). An AxyPrepDNA gel extraction kit was used to purify the PCR products and remove salts and proteins to construct a MiSeq library. The PCR products were also checked by 2% agarose gel electrophoresis before and after gel extraction. DNA sequence degeneracy was described according to the International Union of Pure and Applied Chemistry Conventions: Y = C/T; S = G/C; R = A/G; B = C/G/T; D = G/A/T; H = T/C/A; N = A/G/C/T; W = A/T; and I = inosine. The most degenerated *nifH* primers pairs according to the conserved amino acid sequence were used and the PCR products were examined using 1.5% agarose gel electrophoresis, purified using a Qiagen gel extraction kit (Qiagen, Hilden, Germany), and sequenced on an Illumina (San Diego, CA, USA) MiSeq PE300 platform by Allwegene Genomics (Beijing, China). Raw data were deposited in BioProject under accession numbers PRJNA597572 and PRJNA597574 for 16S rDNA and *nifH*, respectively.

After low quality, ambiguous reads had been filtered and chimeric sequences had been removed using UCHIME^1^ (Edgar et al., 2011), high-quality sequences were clustered into operational taxonomic units (OTUs) at 97% similarity and bacterial taxonomy was assigned phylogenetically using the Ribosomal Database Project (RDP) classifier (Cole et al., 2014). The raw data analysis of the *nifH* gene fragments was carried out in a similar manner to the 16S rRNA high-throughput sequencing and submitted to the Non-Redundant Protein Sequence Database and Nucleotide Sequence Database from National Center for Biotechnology Information (NCBI nr/nt database). Next, α-diversity indices used to estimate bacterial diversity (Shannon and Simpson) and richness (Chao1 and ACE) were calculated based on OTUs using Mothur^2^ (Schloss et al., 2009). Venn diagrams were constructed using Venny^3^ and community sequencing data were subjected to taxonomic diversity analysis using QIIME (Caporaso et al., 2010). Principal co-ordinates analysis (PCoA) was conducted on Bray-Curtis dissimilarity matrices of OTUs at 97% cut-off in R Studio to reveal community-level differences between treatments (McMurdie and Holmes, 2013). Sequencing and data analysis were carried out by Allwegene Genomics (Beijing, China).

### Isolation and Identification of Nodule Bacteria

To isolate nodules, plants were gently uprooted and taken to the laboratory, where root samples were washed thoroughly under running tap water, surface-sterilized in a 3% sodium hypochlorite solution with 0.02% Tween 20 for 3 min, rinsed three times in sterilized distilled water, and dry-blotted onto sterilized filter paper (Lopez-Gomez et al., 2017). The nodules were then ground in a sterilized mortar, streaked onto a Luria-Bertani (LB) agar plate and incubated at 30°C for 2 days or onto a yeast extract mannitol agar (YMA) plate and incubated at 30°C for 5 days. Purified colonies were preserved in 20% (v/v) glycerol at −80°C for long-term storage and in slants for regular use.

Isolated strains were identified according to Singh et al. (2019). Briefly, genomic DNA was isolated using a Wizard® Genomic DNA Purification Kit (Promega) and 16S rRNA was amplified by PCR using 27f (5'-AGAGTTTGATCMTGGCTCAG-3') and 1492r (5'-GGTT-ACCTTGTACGACTT-3') universal primers with an Applied Biosystems PCR system. Amplified PCR products were purified by 1.2% agarose gel electrophoresis (Bio-Rad, Shanghai, China) and sequenced from both directions at Qingke Biotech (Qingdao, China). Sequence annotations were compared to the EzBioCloud database (Yoon et al., 2017).

### Construction of Artificial Culture System for Isolate Nodulation

Two-hundred wild soybean seeds collected from the Yellow River Delta were scarified by immersion in concentrated H_2_SO_4_ (98.3% vol/vol) for 5 min to make the seed coat thinner and more conducive to germination. Then, the seeds were immediately washed with cool running sterile water for 5–10 min and germinated on 1.0% water-agar plates at 25°C in the dark (Lopez-Gomez et al., 2017). Once rooting and germination had occurred after approximately 2 days, the seedlings were transferred to 100 ml of sterile nitrogen-free liquid medium [3 g Ca(NO_3_)_2_•4H_2_O, 0.46 g CaSO_4_, 0.075 g KCl, 0.06 g MgSO_4_•7H_2_O, 0.136 g K_2_HPO_4_•2H_2_O, 0.075 g iron citrate, and 1 ml of trace element solution (2.86 g H_3_BO_3_, 1.81 g MnSO_4_, 0.22 g ZnSO_4_, 0.80 g CuSO_4_•5H_2_O, and 0.02 g H_2_MoO_4_ in 1000 ml ddH_2_O), and 1,000 ml of ddH_2_O] according to Chen and Wang (2011) in 21 grass test tubes (200 × 40 mm) scaffolded with filter paper. Two bacterial isolates were from the Yellow River Delta and one was from the coastal region. For each bacterial isolate, five tubes were used as the experimental group and two as the control group, with three seedlings per tube. After 2 days, the seedlings were inoculated into 1 ml of nitrogen-free liquid medium containing *Sinorhizobium* sp., (c. 10^9^ cell/ml) grown in tryptone-yeast extract (TY) medium (5 g tryptone, 3 g yeast extract, 0.7 g CaCl_2_•2H_2_O, 1,000 ml ddH_2_O, and pH 6.8–7.0) and washed twice in logarithmic phase by suspension and centrifugation in nitrogen-free liquid medium to remove the TY medium nutrients. Plants were grown in a controlled environmental chamber for 2 weeks with a 16/8 h light-dark cycle, 23/18°C day-night temperature, and 55/65% relative humidity. Control plants were cultivated in the same nutrient solution without *Sinorhizobium* sp., inoculation.

### Statistical Analysis

Results are presented as the mean ± standard deviation of three independent experiments. Statistical differences were determined by one-way analysis of variance followed by Tukey’s test in SPSS version 17.0 (IBM, Armonk, NY, USA), with *p* < 0.05 considered significant.

## Results and Discussion

### Bacterial Community Composition in the Root Zone of Saline-Alkali Soils

**Figure 1:**
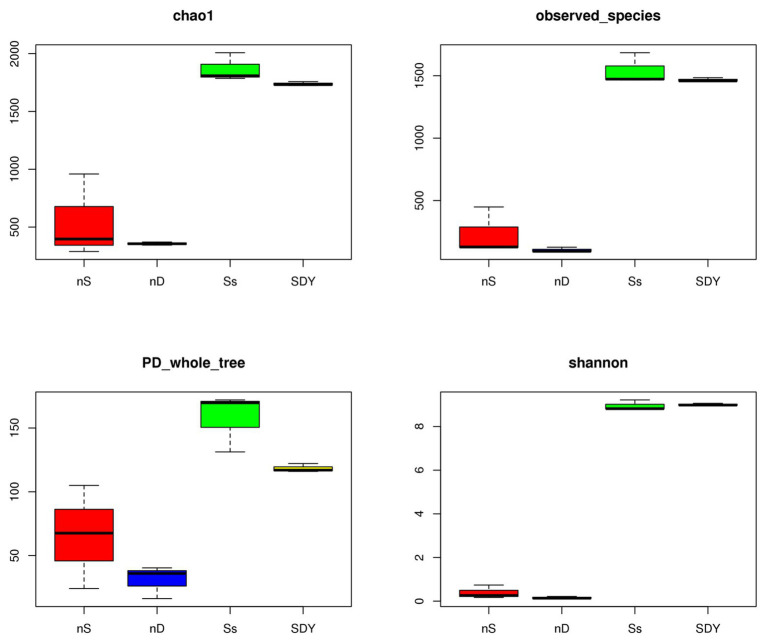
Alpha diversity indices of 16S rRNA sequences in the two nodule samples and two root zone samples from wild soybean.

To investigate the effect of saline-alkali soils on the root and nodule microbiomes of wild soybean, we analyzed the root zone microbiome of wild soybean grown in two saline-alkali soil types from the Yellow River Delta near to Dongying city (SDY) and coastal soil (Ss). A total of 1,528 ± 39 and 1,797 ± 246 OTUs were obtained from the clean sequences from the SDY and Ss root zones, respectively (Supplementary Table S1), whereas only 358 ± 209 and 164 ± 10 OTUs were obtained from the nodules of wild soybean grown in these regions, respectively. We found that the barren sand root zone carried more OTUs than the fertile soil; however, our previous study identified 191 ± 13 OTUs in nodules using PacBio’s circular consensus sequencing for full-length bacterial 16S rRNA (Zheng et al., 2020), similar to the 164 ± 10 OTUs obtained here. The dataset was rarefied to an even sequencing depth of 20,000 sequences and 2,423 bacterial OTUs were identified. The Shannon and Simpson diversity indices and Chao1 richness estimator for the 16S V5-V7 replicon sequencing of these samples are shown in Figure 1 and Supplementary Table S2, while the Venn diagram is shown in Supplementary Figure S1.

Although we found no significant difference in the number of clean sequences between the root zones and nodules (Supplementary Table S3), the number of OTUs did differ significantly, with the SDY nodules having around one-tenth the number of OTUs of the SDY root zone. In addition, there were approximately twice as many OTUs in the nodules of the Ss samples than in the SDY samples.

**Figure 2:**
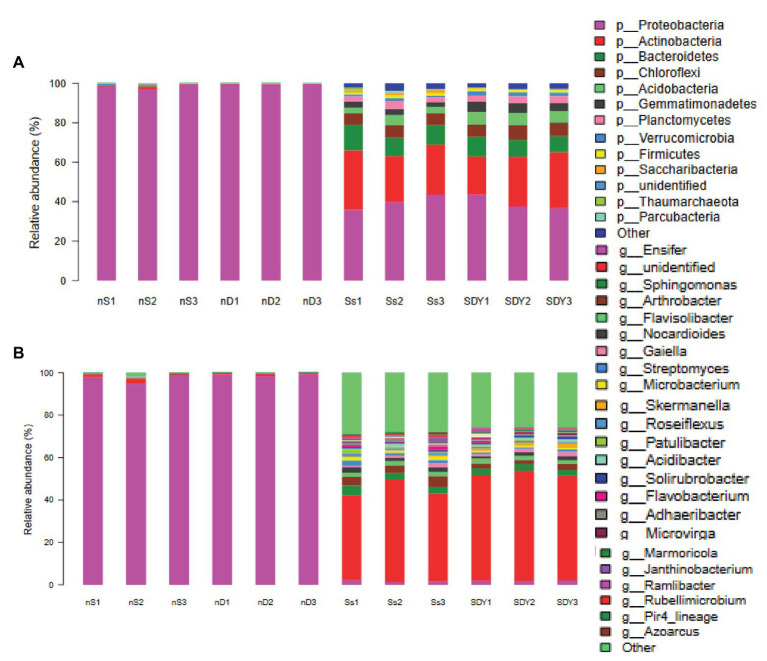
Bacterial community composition related to the root of wild soybean. Relative abundance at the **(A)** phylum and **(B)** genus levels.

Overall, 99.67 ± 0.34% of the sequences obtained from the root zone of wild soybean from Ss samples were assigned to a bacterial taxonomy, compared to 95.89 ± 1.63% of those in the SDY samples. The nine most abundant phyla (>1% sequences) comprised 95.42 ± 0.33 and 96.75 ± 0.24% of the root zone community in Ss and SDY samples, respectively (Supplementary Table S3), with 39.67 ± 2.87% and 39.67 ± 3.09% of the sequences belonging to Proteobacteria (Supplementary Table S4; Figure 2A). The second most abundant phylum was Actinobacteria (26.00 ± 2.94 and 24.00 ± 3.74% in Ss and SDY root zones, respectively), while other phyla identified in the root zones included Bacteroidetes (10.67 ± 1.70 and 9.00 ± 0.82%), Chloroflexi (6.00 ± 0.05 and 6.67 ± 0.47%), Acidobacteria (3.67 ± 0.90 and 6.00 ± 0.02%), Gemmatimonadetes (2.67 ± 0.47 and 4.67 ± 0.47%), Planctomycetes (3.00 ± 0.83 and 3.67 ± 0.47%), and Firmicutes (1.30 ± 0.50 and 1.00 ± 0.01%). The Ss root zone samples also included Saccharibacteria (1.33 ± 0.47%), which only accounted for significantly less than 0.20 ± 0.00% of the root zone abundance in SDY samples. Another significant difference in relative abundance was observed for Verrucomicrobia (0.90 ± 0.14 and 2.00 ± 0.00% in Ss and SDY root zones, respectively) as well as Acidobacteria, which could be due to differences in pH between the root zones of wild soybean grown in coastal sand (pH 6.5) and saline-alkali soil from the Yellow River Delta (pH 7.6). At the genus level, *Sinorhizobium/Ensifer*, which is the dominant genus in nodules, accounted for just 1.59 ± 0.40 and 1.87 ± 0.20% of the species in Ss and SDY root zones, respectively (Supplementary Table S5; Figure 2B). The relative microbial abundance in six root zone samples and six nodule samples at the order and family levels, as determined by 16S Illumina sequencing, is shown in Supplementary Figure S2.

### Identification of Nodule Bacterial Community Composition

The 16S rRNA sequences amplified from the uncultured root nodule samples were assigned to 14 phyla and an overview of the dominant phyla and genera is provided in Supplementary Tables S4 and S5. Based on relative abundance, the most common phyla (>0.1%) in the coastal nodule samples (nS) and Yellow River Delta nodule samples (nD), respectively, were Proteobacteria (98.458 ± 0.045 and 99.703 ± 0.002%), Actinobacteria (0.533 ± 0.340 and 0.167 ± 0.047%), Bacteroidetes (0.177 ± 0.158 and 0.037 ± 0.017%), Chloroflexi (0.123 ± 0.122 and 0.047 ± 0.009%), Acidobacteria (0.113 ± 0.092 and 0.043 ± 0.012%), and Gemmatimonadetes (0.117 ± 0.095 and 0.043 ± 0.009%), indicating that nS samples displayed a higher diversity and proportion than nD samples. As predicted, the most prominent Proteobacteria were *Sinorhizobium/Ensifer* (97.330 ± 1.701 and 98.661 ± 0.471%); however, the proportion differed (85.67 ± 6.29%) from PacBio’s circular consensus sequencing for full-length bacterial 16S rRNA gene in nD samples (Zheng et al., 2020).

To investigate the diversity of bacteria related to saline tolerance and plant growth promotion, we focused on the minor dominant genera beyond *Sinorhizobium/Ensifer*. Besides Proteobacteria, Actinobacteria was the most abundant phylum in the two types of nodule samples. *Microbacterium*, *Arthrobacter*, *Nocardioides*, and *Streptomyces* were the most predominant Actinobacteria in nS samples, with relative abundances of over 0.03%, while *Blastococcus* and *Patulibacter* had relative abundances of over 0.005%. In the α-proteobacteria, *Sphingomonas* (60.0, 68.3% in Sphingomonadaceae) accounted for 0.086 ± 0.009 and 0.016 ± 0.004% of all bacteria in the nS and nD samples, respectively, while *Variibacter* (77.0, 67.1% in Xanthobacteraceae) accounted for 0.015 ± 0.006 and 0.013 ± 0.004%. Other identified α-proteobacteria genera included *Devosia*, *Pedomicrobium*, and *Microvirga* (Supplementary Table S5). In the γ-proteobacteria, some OTUs were only identified as belonging to the Enterobacteriaceae family (38.3 ± 14.5 and 31.7 ± 21.3% in the nS and nD samples, respectively), which have been reported to be PGPR (Agler et al., 2016). *Pseudomonas* was the predominant genus identified, accounting for 0.032 ± 0.008 and 0.027 ± 0.007% in Ss and SDY root zones of all bacteria, while *Acinetobacter* and *Pseudoxanthomonas* had relative abundances of >0.01%. *Flavobacterium* (24.0 ± 10.7%) was the predominant Bacteroidetes genus in nS samples, accounting for 0.042 ± 0.010% of all bacteria, but only making up 2.0 ± 1.5% of *Bacteroidetes* in nD samples. *Flavisolibacter* was another dominant Bacteroidetes genus (Supplementary Table S5). *Bacillus* (59.5%), *Paenibacillus* (18.6%), *Halobacillus* (21.4%), and *Lysinibacillus* (10.4%) were the most predominant Firmicutes genera in nS samples, constituting 0.022 ± 0.005, 0.008 ± 0.003, 0.004 ± 0.002, and 0.004 ± 0.001% of all bacteria, respectively (Supplementary Table S5).

### Comparison of Bacterial Community Composition in Different Soil Samples

Although Ss and SDY samples displayed similar proportions of Actinobacteria (26:24%), we found that levels were more than three times higher in nodule samples, suggesting that Actinobacteria may be involved in saline tolerance in the rhizosphere of wild soybean grown in coastal regions (Bhatti et al., 2017). In α-proteobacteria, nS samples had more than five times as many *Sphingomonas* species than nD samples; however, it has previously been reported that these root zones display similar proportions of *Sphingomonas* species (Menon et al., 2019). Similarly, nS samples also harbored a higher proportion of the γ-proteobacteria Enterobacteriaceae and *Pseudomonas* families, which have also been reported as PGPR (Agler et al., 2016; Chu et al., 2019). Despite thorough cleaning before environmental DNA extraction, the surface of the nodules contained some rhizosphere bacteria from the root zone. Although the salinity of the coastal sand was lower than that of fertile soil from the Yellow River Delta, its sandy characteristics result in a lower water holding capacity, meaning that the relative salinity of the sand was actually higher than that of the soil.

The nodules had similar proportions of γ-proteobacteria and thus could be used as a control to compare changes in the ratio of different bacteria. We found that the pH 7.9 alkali soil had a higher relative abundance of Acidobacteria than the pH 6.5 coastal sand, indicating that Acidobacteria may play an important role in the degradation of plant residues due to a high organic matter content (Kielak et al., 2016). Moreover, the relative abundance of Acidobacteria in nS samples was nearly three times that in nD samples, suggesting that root exudates provide an ecological niche for the enrichment of Acidobacteria as symbionts. To our knowledge, this is the first study to investigate the relationship between the nodule and root zone microbiomes of wild soybean to understand PGPR in saline-alkali soils.

### Beta Diversity Analysis of 16S rRNA Sequences in Root Zone and Nodule Samples

**Figure 3:**
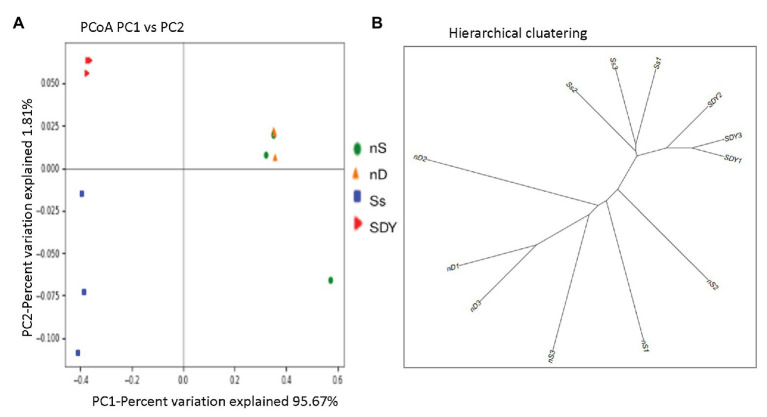
Unconstrained principal coordinate analysis (PCoA) of weighted U*nif*rac distances for root zone and nodule samples **(A)**. Hierarchical clustering analysis **(B)**.

Analysis of bacterial community composition revealed that the microbiomes of the coastal sand root zone or nodule samples were richer and phylogenetically more diverse than those of the saline-alkali soil in the Yellow River Delta. Therefore, we quantified the major components driving differences between samples (β-diversity) using unconstrained principal coordinates analysis (PCoA) on weighted UniFrac distances. We found a clear separation along axis 1 (explaining 95.67% of the overall variation) and confirmed the general pattern that root zones and nodules harbor distinct microbiomes (Figure 3A). Axis 2 explained 1.81% of the overall variation and mainly separated the root zone samples, with no obvious clustering observed between wild soybean grown in the two soil types, suggesting that growth conditions have negligible effects on β-diversity. Hierarchical clustering was performed using the unweighted pair group method with arithmetic mean to understand the relationship between these samples, indicating that the two root zones had similar microbiomes but formed two branches, as shown in Figure 3B.

**Figure 4:**
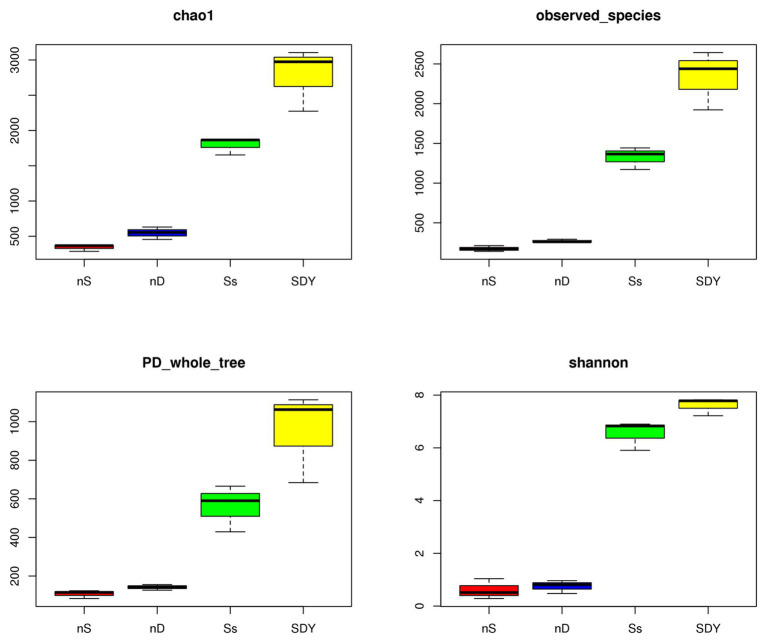
Alpha diversity indices of *nifH* sequences in the two nodule samples and two root zone samples from wild soybean.

To further understand the relationship between these samples, we performed principal component analysis (PCA) based on OTU relative abundance (Supplementary Figure S3). We found that each sample type formed a cluster, indicating reliable biological replication and that the bacterial composition statistic correctly reflected the microbiomes of the nodules or root zone of wild soybean grown in coastal sand or saline-alkali soil from the Yellow River Delta. Moreover, this analysis revealed that soil type predominantly shaped the assembly of the rhizosphere microbiome (Liu et al., 2019), demonstrating that rhizobial evolution in different geographic locations is related to soil type, altitude, and spatial effects (Zhao et al., 2014).

### Composition Analysis of *nifH* Genes in Root Zones and Nodules

The most degenerated *nifH* primer pairs were used to amplify the environmental DNA extracted from the nodules and root zone, following which the Illumina sequencing data were summarized based on the PCR products (Supplementary Table S6). The α-diversity indices for *nifH* replicon sequencing of two root zone and nodule samples are listed in Figure 4 and Supplementary Table S6. A total of 67,017 ± 4,505 and 68,701 ± 6,364 clean sequences were obtained from nS and nD nodule samples, whereas around twice as many (131,744 ± 27,556 and 148,306 ± 22,497) were obtained from the Ss and SDY root zone samples (Supplementary Table S7). OTU identification revealed that there were approximately eight times as many OTUs in the root zones as in the nodules, indicating that root zone diversity was much richer (Supplementary Table S7). The length of the *nifH* PCR products varied from 240 to 480 bp, indicating that the degenerated *nifH* primer pairs were able to amplify as many different *nifH* genes as possible (Supplementary Figure S4).

**Figure 5:**
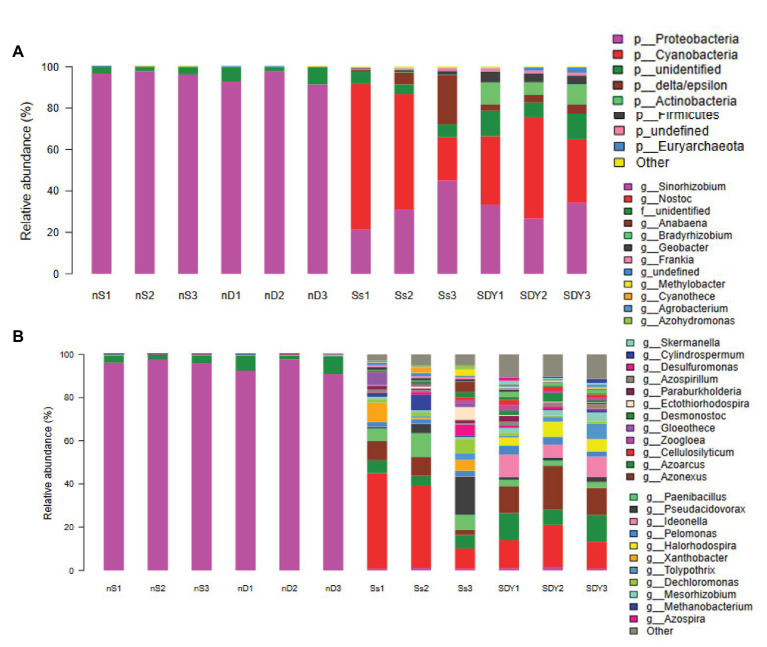
*nifH* composition related to the root of wild soybean. Relative abundance at the **(A)** phylum and **(B)** genus levels.

The Venn diagram of *nifH* OTUs identified 165 shared OTUs in the four different soybean root-related microbiomes (Supplementary Figure S5), while the associated α-diversity indices for *nifH* replicon sequencing are shown in Supplementary Table S7. In the nodules, 96.657 ± 0.943 and 93.342 ± 2.645% of sequences were identified as *Sinorhizobium/Ensifer* (Supplementary Table S8), but may not all have been from the same single species. For instance, a previous study isolated and identified *Ensifer fredii*, *Ensifer morelense*, *Rhizobium radiobacter*, and a putative novel *Rhizobium* species from the root nodule of wild soybean in northwest China, all of which formed a single lineage related to *E. fredii* in *nodA* and *nifH* gene phylogenies, suggesting that symbiotic genes are laterally transferred between species (Zhao et al., 2014). In the root zones, the relative abundance (>1%) of *nifH* genes in Ss and SDY samples was as follows: Cyanobacteria (49.000 ± 20.607 and 37.667 ± 8.055%), Proteobacteria (32.323 ± 9.843 and 31.333 ± 3.091%), unidentified (5.672 ± 0.473 and 10.333 ± 2.357%), delta/epsilon (10.000 ± 7.204 and 3.667 ± 0.471%), Firmicutes (0.668 ± 0.231 and 4.104 ± 0.393%), Actinobacteria (0.238 ± 0.047 and 9.000 ± 2.160%), and Euryarchaeota (0.100 ± 0.007 and 1.733 ± 1.159%; Supplementary Table S9; Figure 5), respectively. In Cyanobacteria, *Nostoc* and *Cylindrospermum* were the predominant genera in Ss or SDY samples (Supplementary Table S10), respectively, while *Bradyrhizobium* (32.7 ± 4.7%) and *Methylobacter* (17.7 ± 6.2%) were the predominant Proteobacteria and *Frankia* was the predominant Actinobacteria in both root zones. In Firmicutes, *Cellulosilyticum*, *Paenibacillus*, *Clostridium*, and *Bacillus* were identified to contain *nifH* genes, with almost half of all *Paenibacillus* isolates having been reported to fix nitrogen in soils (Xie et al., 2014). The relative abundance of *nifH* gene fragments from Illumina sequencing at the order, family, and species level are explored in detail in Supplementary Figure S6.

**Figure 6:**
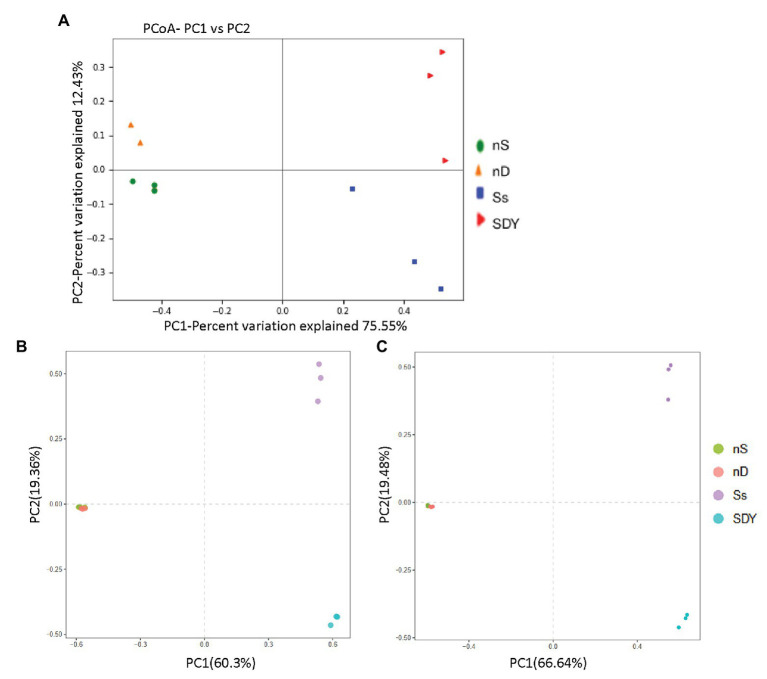
Principal coordinate analysis (PCoA) of weighted U*nif*rac distances based on *nifH* operational taxonomic units (OTUs) **(A)** and principal component analysis (PCA) based on OTU relative abundance **(B)** and diff-OTUs relative abundance **(C)** in root zone and nodule samples.

PGPR can fix nitrogen *via* non-symbiotic bacteria, thus enabling a biologically inactive form of nitrogen to be readily used by organisms (Singh et al., 2020; Soares et al., 2020). Since diverse microbial communities use this process to fulfill their nitrogen demands (Liu et al., 2012), the phylogenetic diversity of *nifH* genes, a molecular marker of nitrogen fixation (Gaby et al., 2018), has been examined extensively in the microbiota of various plants and invertebrates, including humans (Yamada et al., 2007; Xiao et al., 2010; Liu et al., 2012; Igai et al., 2016). At the phylum level, we found that Cyanobacteria accounted for 49.0% of the sequences identified in coastal sand, but only 37.7% of those in the Yellow River Delta samples. Although unexpected, this result was consistent with a previous study which found that Geobacter and Cyanobacteria are important functional components of the nitrogen-fixing community in agricultural soil based on *nifH*-RNA transcriptomic sequencing analysis (Calderoli et al., 2017). We found no difference in the relative abundance of *nifH* sequences belonging to proteobacteria in the two root zones (approximately 32 and 31% in Ss and SDY root zones, respectively); however, the ratio of *nifH* belonging to α-, β-, γ-, and δ-proteobacteria did differ. Previously, the γ-proteobacteria *Pseudomonas* were isolated as the main nitrogen-fixing bacterial isolates from three rhizosphere soil samples taken from mangrove plants in the Dongzhaigang National Mangrove Nature Reserve of China (Liu et al., 2012). Using a soil DNA extraction and PCR-cloning-sequencing approach the study analyzed 135 clones and identified 27 unique *nifH* sequence phylotypes, most of which were closely related to sequences from uncultured bacteria; however, the other seven were identified as nitrogen-fixing α-proteobacteria (*Bradyrhizobium*, *Rhodospirillum*, and *Rhodobacter*) or *Archaea* (Liu et al., 2012). In this study, significant differences in *nifH* relative abundance were observed between Firmicutes (0.67 and 4.10%), Actinobacteria (0.24 and 9.00%), and one Archaea phylum Euryarchaeota (0.10 and 1.73%) in Ss and SDY samples, respectively. Conversely, no significant difference was observed in the nodule samples due to the overwhelming relative *nifH* abundance, which may be the result of host selection in different environments.

### Beta Diversity Analysis of *nifH* Sequences in Root Zone and Nodule Microbiomes

*nifH* composition analysis revealed that *nifH* sequence diversity appeared to be richer in nD and SDY samples (Figure 5). To understand this similarity, we quantified the major components driving β-diversity using unconstrained PCoA on weighted UniFrac distances. We found a clear separation along axis 1 that explained 75.55% of all variation and confirmed a general pattern with root zones and nodules harboring distinct *nifH* genes (Figure 6A). Conversely, axis 2 explained 12.4% of the overall variation and mainly separated the root zone samples. To further understand the relationship between these samples, we performed PCA on OTU relative abundance (Figures 6B,C). Each sample type formed a cluster, indicating that biological replication was reliable and that the bacterial composition statistic correctly reflected the *nifH* gene set in the nodules and root zones of wild soybean grown in coastal sand or saline-alkali soil in the Yellow River Delta.

### Isolated Members of Wild Soybean Nodule Microbiota

**Figure 7:**
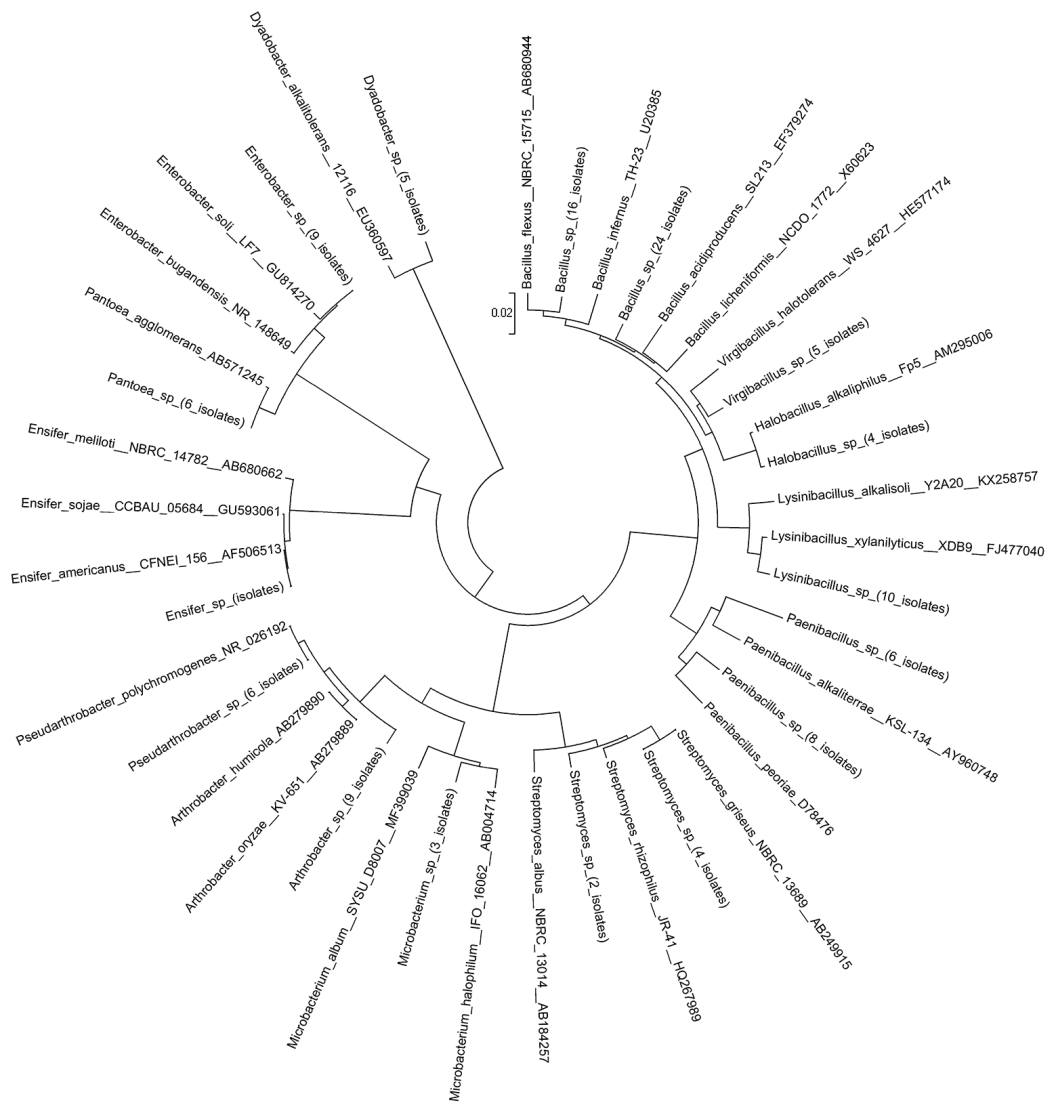
Phylogenetic tree of 16S rRNA gene sequences showing the relationship between the representative and reference isolates. The neighbor-joining (NJ) tree was derived from a 16S rRNA gene sequence distance matrix (Kimura two parameter).

To identify potential PGPR from the rhizosphere and endophytes of wild soybean roots grown in saline-alkali soil, we isolated bacteria from wild soybean nodules. A total of 277 cultured bacteria were characterized, including 180 from nS samples and 97 from nD samples (Figure 7). The α-proteobacteria genus *Sinorhizobium/Ensifer* dominated the cultured bacteria and accounted for 81 nS isolates and 38 nD isolates (43.0% of all isolates), the majority of which were able to grow in 2% NaCl + LB medium. A previous study investigated the diversity and biogeography of *G. soja*-nodulating rhizobia by characterizing 155 nodule isolates from seven sites in northwest China by 16S rRNA PCR-RFLP and the sequence analysis of multiple core genes (16S rRNA, *recA*, *atpD*, and *glnII*). Among the isolates, 80 were *Ensifer fredii*, 19 were *Ensifer morelense*, 49 were *Rhizobium radiobacter*, and seven were putative novel *Rhizobium* species (Zhao et al., 2014). In this study, we identified all isolates as *Ensifer americanum* based on 16S rRNA, while the γ-proteobacteria *Enterobacter* (nine isolates) and *Pantoea* (six isolates) were isolated from nS and nD samples, respectively.

Of the Firmicutes, *Bacillus* was the second most predominant bacterial genus, accounting for 40 nS isolates and 41 nD isolates, representing 29.2% of all bacteria and 71.1% all Firmicutes (81/114). *Bacillus* sp., has been reported to possess versatile traits that protect plant against diverse abiotic stresses, including heat, cold, and freezing (Tiwari et al., 2017). Indeed, *Paenibacillus* (14 isolates) have been reported to provide their host with multiple benefits, including nitrogen fixation, phosphate solubilization, and biocontrol (Grady et al., 2016). *Lysinibacillus* (10 isolates), *Virgibacillus* (five isolates), and *Halobacillus* (four isolates) were also isolated and identified from the nS and nD samples.

Actinobacteria and Bacteroidetes accounted for 8.7 and 1.8% of all isolates, respectively. We identified four Actinobacteria genera (24 isolates) with more than one representative isolate, including *Arthrobacter* (nine isolates), *Streptomyces* (six isolates), *Pseudarthrobacter* (six isolates), and *Microbacterium* (three isolates), which were mainly from nS samples, with the exception of *Arthrobacter*. Conversely, only one Bacteroidetes genus *Dyadobacter* (five isolates) was isolated from nS samples. Our previous study at the species level identified four genera with high relative abundance: *Enterobacter*, *Chryseobacterium*, *Stenotrophomonas*, and *Flavobacterium* (Zheng et al., 2020); however, only *Enterobacter* spp. were isolated in this study, which have been reported to be microsymbionts of *G. soja* (Zhao et al., 2014).

### Functional Study of Wild Soybean Root Microbiota

**Figure 8:**
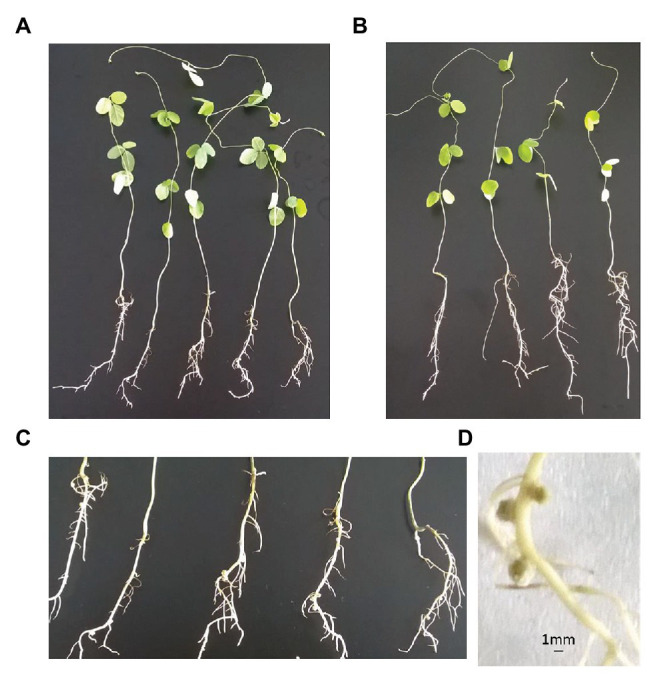
Wild soybean nodule formation **(A)** when inoculated with *Sinorhizobium/Ensifer* and **(B)** without inoculation with *Sinorhizobium/Ensifer*. **(C)** Root nodule formation when inoculated. **(D)** Nodule size and shape.

To investigate the effect of the microbiota isolates on wild soybean growth and nodule formation, we developed a sterile hydroponics cultivation method using nitrogen-free medium and evaluated its potential to examine plant-microbiota interactions. When inoculated with *Sinorhizobium/Ensifer* isolates, nodule formation was observed in the wild soybean seedlings with less than 2 weeks of cultivation and plant biomass was slightly higher than in seedlings without inoculation, with approximately half as many nodules as formed in the wild (Figure 8; Supplementary Figure S7).

Our experimental hydroponics cultivation system enabled us to test the effects of microbiota members isolated from root nodules on plant growth and thus represents a possible approach to advance our functional understanding of the root microbiome. Consequently, we believe that our study is a pioneering example of novel laboratory research and future studies with different isolation media and growth conditions will allow us to broaden the reference stock to investigate PGPR in saline-alkali soil.

## Data Availability Statement

The datasets generated for this study can be found in the NCBI BioProject under accession numbers PRJNA597572 and PRJNA597574.

## Author Contributions

YY, RS, and CZ generated ideas, directed the work, conducted experiments, and wrote and edited the manuscript. YY and CJ isolated and characterized the initial bacterial isolates, including sequence analysis. CM and SM prepared some figures. CM, CJ, YL, and CZ obtained funding. All coauthors reviewed the manuscript. All authors contributed to the article and approved the submitted version.

### Conflict of Interest

The authors declare that the research was conducted in the absence of any commercial or financial relationships that could be construed as a potential conflict of interest.

## Footnotes

^1^http://drive5.com/uchime

^2^https://www.mothur.org/

^3^http://bioinfogp.cnb.csic.es/tools/venny/index.html

## Supplementary Material

The Supplementary Material for this article can be found online at:

https://www.frontiersin.org/articles/10.3389/fmicb.2020.523142/full#supplementary-material
