# Supplementary material for: Nodule and Root Zone Microbiota of Salt-Tolerant Wild Soybean in Coastal Sand and Saline-Alkali Soil
Source: Front Microbiol. 2020 Sep 22;11:2178. doi: 10.3389/fmicb.2020.523142 (PMC7536311; doi:10.3389/fmicb.2020.523142)
Supplement: Supplementary file 1 [file Data_Sheet_1.docx]

**Title: Nodule and Root Zone Microbiota of Salt-tolerant Wild Soybean in Coastal Sand and Saline-alkali Soil**

Yingjie Yang^a^, Lei Liu^b^, Raghvendra Pratap Singh^c^*, Chen Meng^a^, Siqi Ma^a^, Changliang Jing^a^, Yiqiang Li^a^ and Chengsheng Zhang^a^*

**Affiliations:**

^a^Marine Agriculture Research Center, Tobacco Research Institute, Chinese Academy of Agricultural Sciences, Qingdao, Shandong 266101, China. YJY: yingjieyoung@sina.com; CM: mengchen01@caas.cn; CLJ: jingchangliang@caas.cn; CSZ: zhangchengsheng@caas.cn; YQL: liyiqiang@caas.cn.

^b^Bureau of Agriculture and Rural Affairs of Laoshan District, Qingdao, Shandong 266061, China. LL: liulei72288@163.com

^c^Department of Research & Development, Biotechnology, Uttaranchal University, Dehradun 248007, India. RPS: rpsingh@uttaranchaluniversity.ac.in

***corresponding authors:** Raghvendra Pratap Singh and Chengsheng Zhang

**Corresponding E-mail:** rpsingh@uttaranchaluniversity.ac.in (R.P. Singh); zhangchengsheng@caas.cn (C.S. Zhang)

Legends of supplementary materials

Fig. S1. the Venn map of 16S Illumina sequencing of six root zones samples and six nodules under saline fields

Fig. S2. the relative abundance of 16S Illumina sequencing of six root zones samples and six nodules under saline fields at order level (2A) and family level (2B)

Fig. S3. Principal Component Analysis (PCA analysis) based on 16S OTU (A) and based on 16S diff-OTU (B).

Fig. S4. Tags distribution of 16Sr RNA V5-V7 regions and nifH gene fragments

Fig. S5. the Venn map of nifH gene fragments Illumina sequencing of six root zones samples and six nodules under saline fields

Fig. S6 the relative abundance of nifH gene fragments Illumina sequencing of six root zones samples and six nodules under saline fields at order level (6A), family level (6B), species level(6C)

Fig. S7 G.soja obtained from the Yellow River Delta (Dongying city) (7A) and Shilaoren coastal region in Qingdao (7B)

Table S1 Sequencing data of 16S V5-V7 region by Illumina sequencing platform

Table S2 the alpha diversity index of 16S V5-V7 region replicon sequencing of two root zone and two nodules of wild soybean grown in saline-alkali soil.

Table S3 The relative abundance of 16S rRNA in root zone of wild soybean

Table S4 The relative abundance of 16S rRNA in the nodules of wild soybean at phylum level

Table S5 The relative abundance of 16S rRNA in the nodules of wild soybean at genus level

Table S6 Sequencing data of *nifH* gene fragments by Illumina sequencing platform

Table S7 the alpha diversity index of *nifH* replicon sequencing of two root zone and two nodules of wild soybean grown in saline-alkali soil.

Table S8 the relative abundance of *nifH* in nodules at phylum level

Table S9 the relative abundance of *nifH* in root zones at phylum level

Table S10 the relative abundance of *nifH* in root zones at genus level


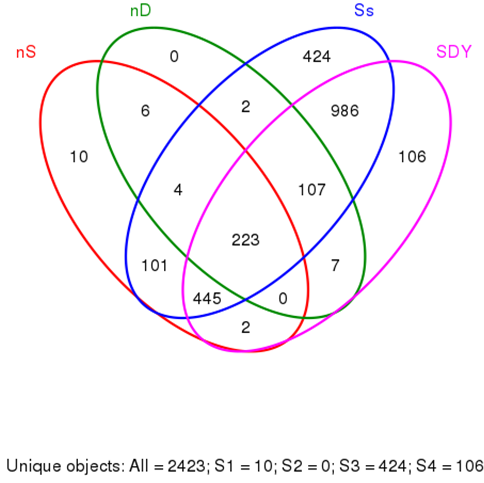


Fig. S1 the Venn map of 16S Illumina sequencing of six root zones samples and six nodules under saline fields

2A


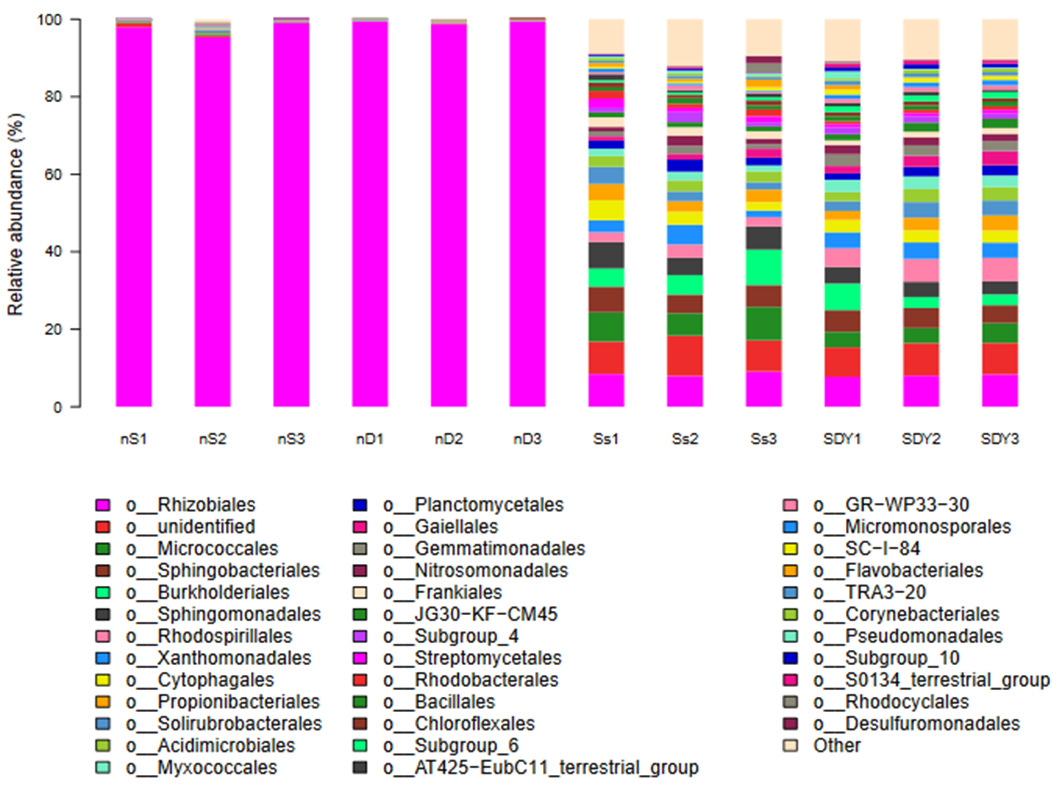


2B


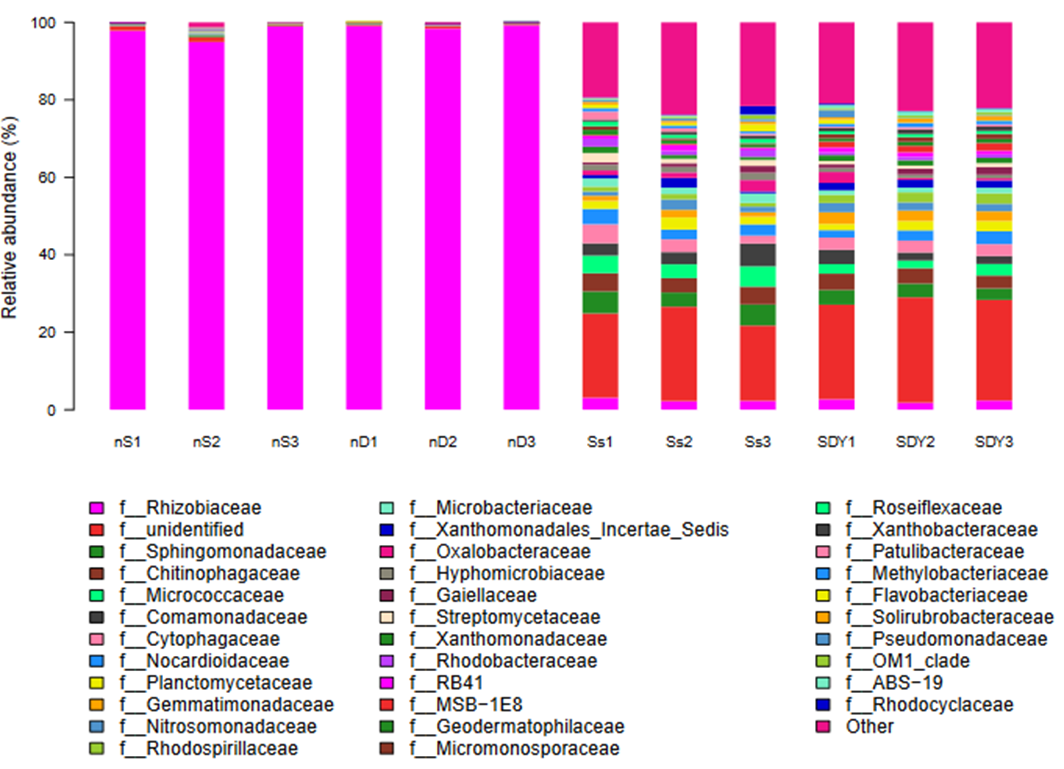


Fig. S2 the relative abundance of 16S Illumina sequencing of six root zones samples and six nodules under saline fields in the order level (2A) and family level (2B).


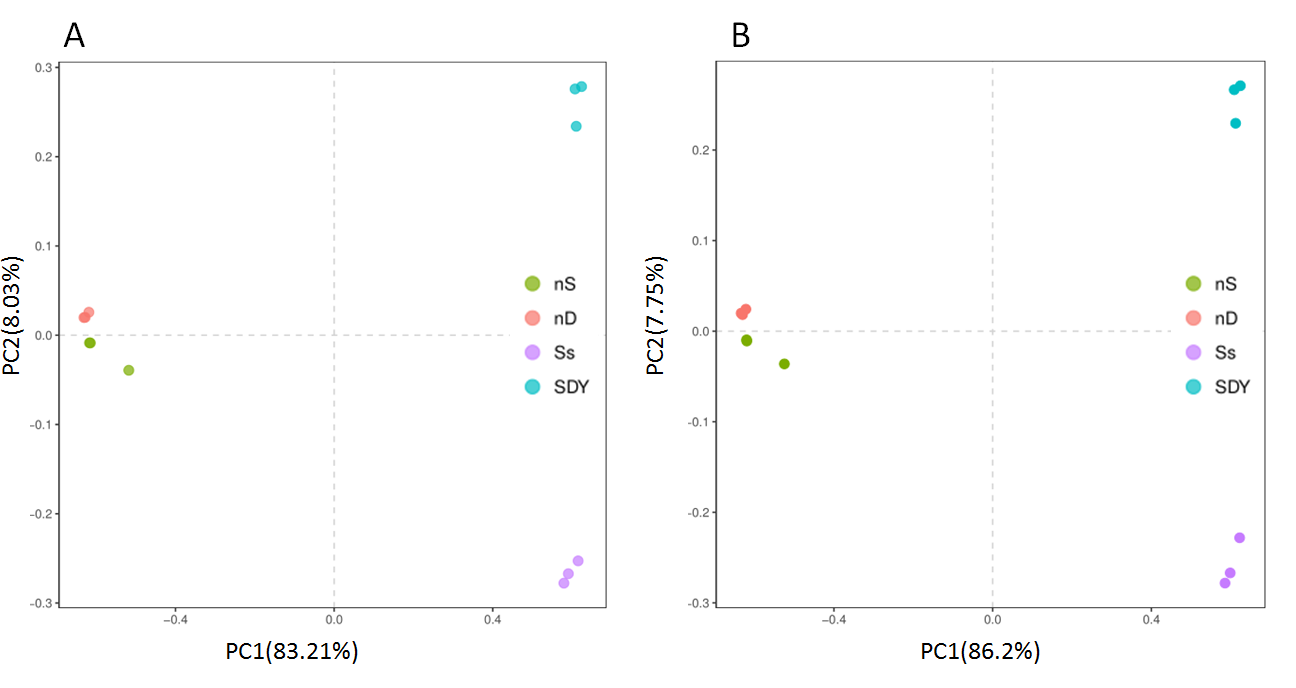


Fig. S3 Principal Component Analysis (PCA analysis) based on 16S OTU (A) and based on 16S diff-OTU (B).


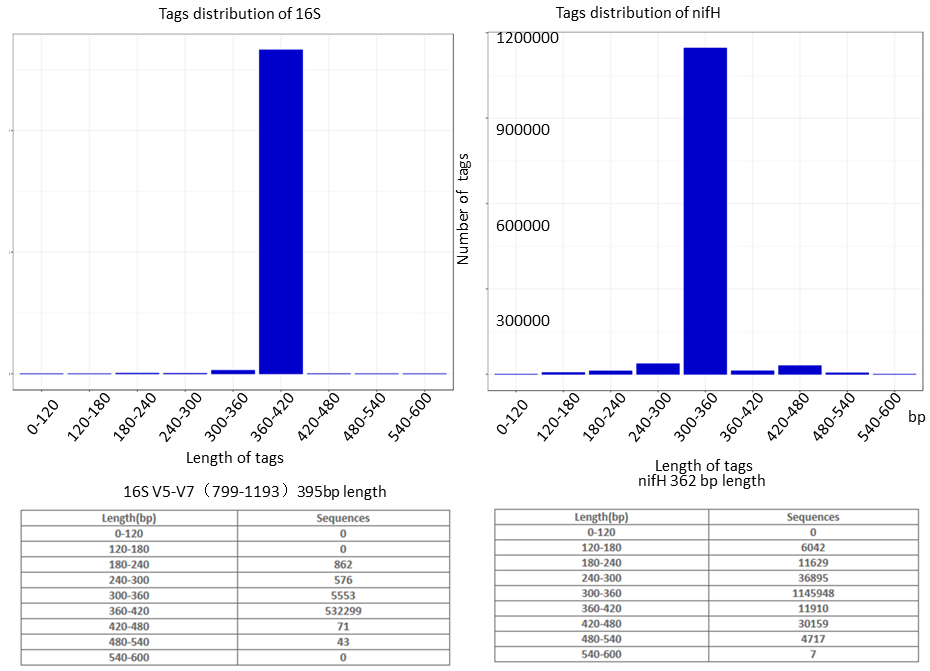


Fig. S4 Tags distribution of 16Sr RNA V5-V7 regions and nifH gene fragments.


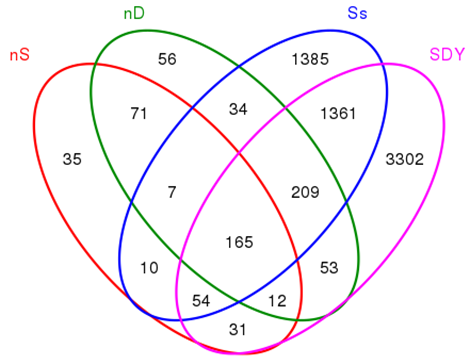


Fig. S5 the Venn map of *nifH* gene fragments Illumina sequencing of six root zones samples and six nodules under saline fields

Fig. S6A


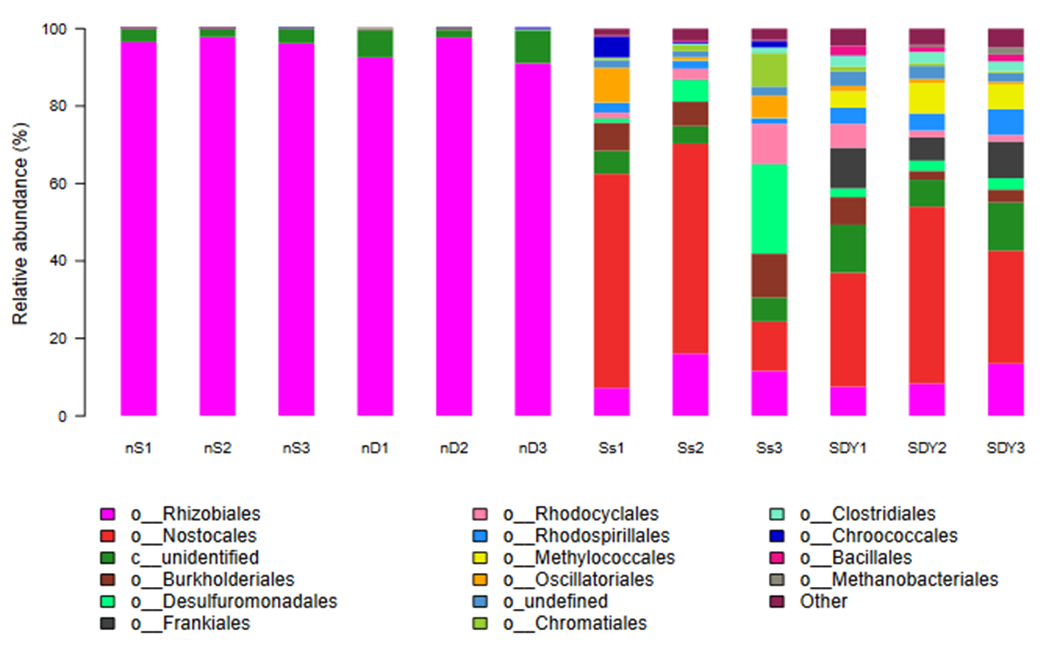


Fig. S6B


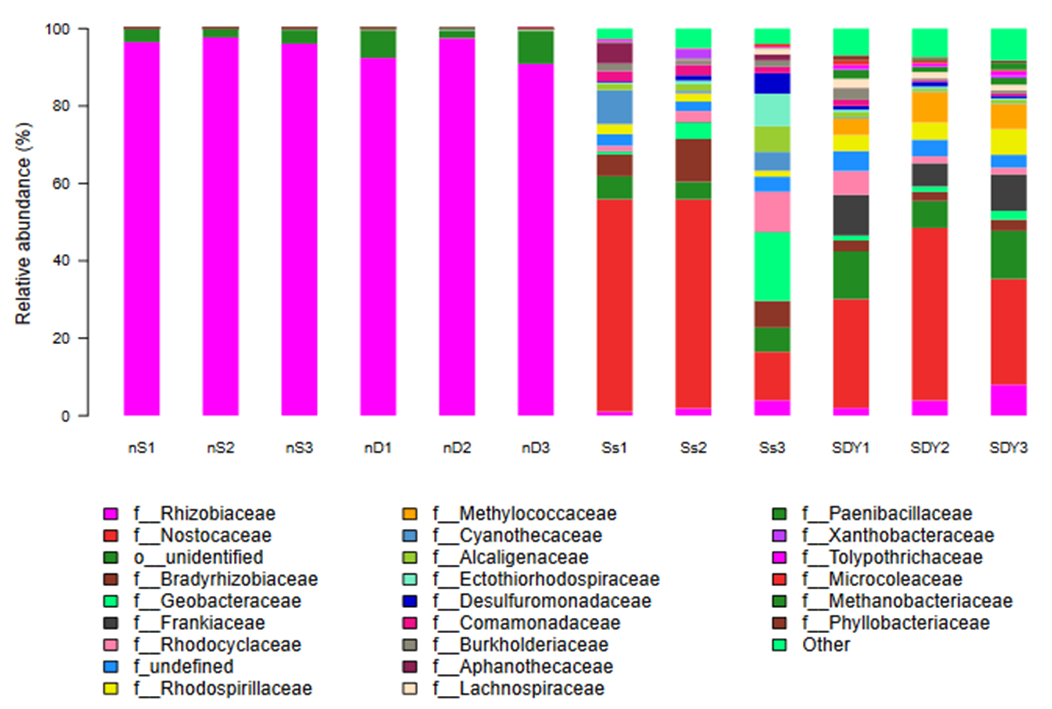


Fig. S6C


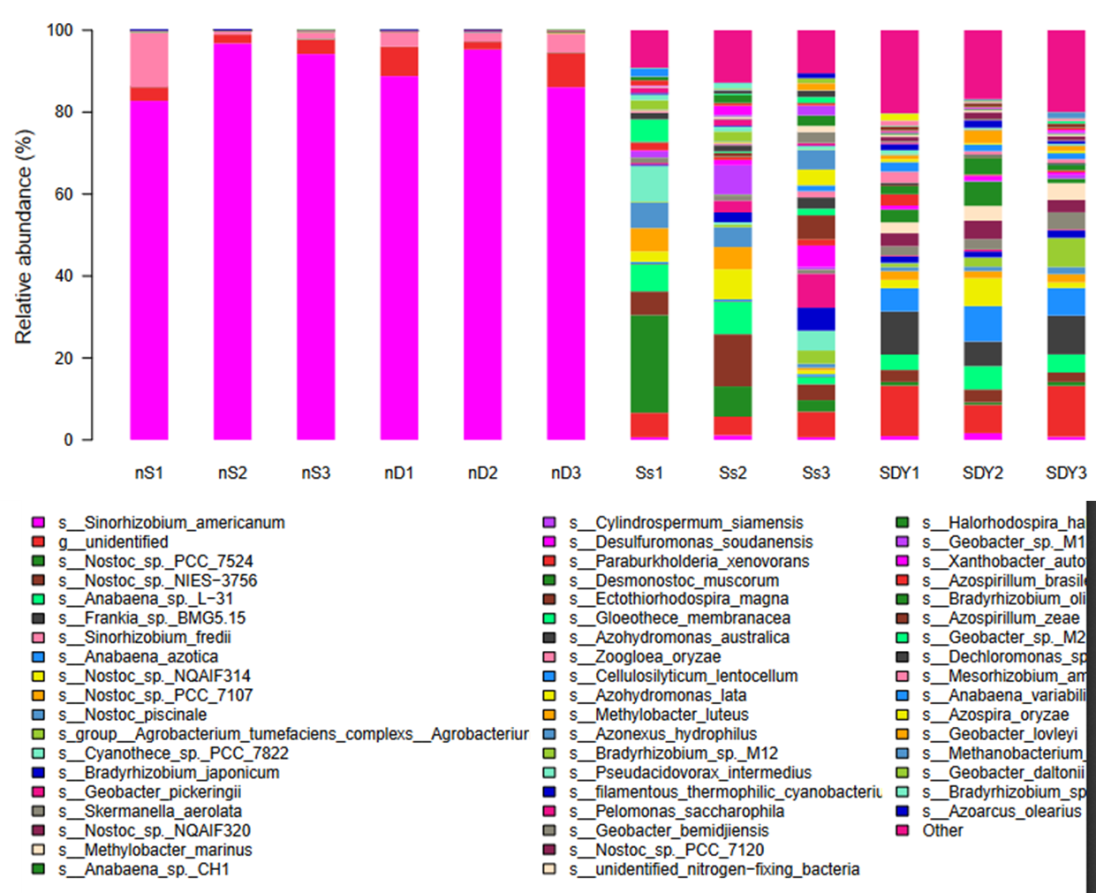


Fig. S6 the relative abundance of *nifH* gene fragments Illumina sequencing of six root zones samples and six nodules under saline fields in the order level (6A), family level (6B), and species level (6C).

Fig. S7A


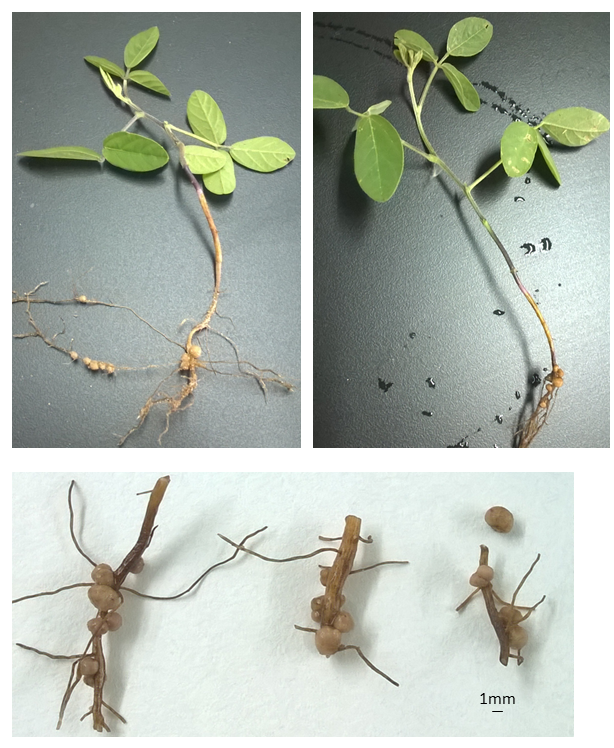


Fig. S7B


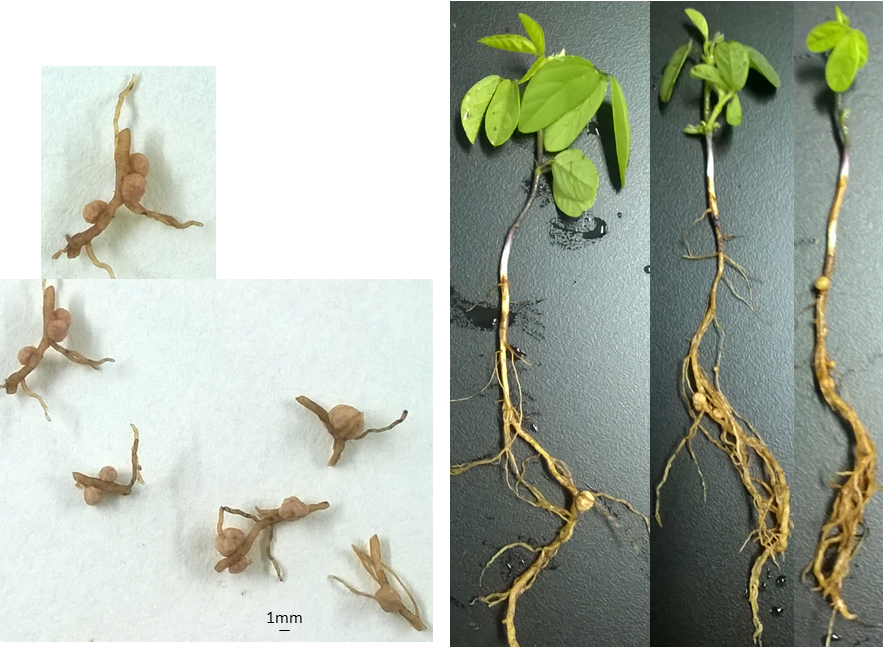


Fig. S7 *G.soja* obtained from the Yellow River Delta (Dongying city) (7A) and Shilaoren coastal region in Qingdao (7B)

Table S1 Sequencing data of 16S V5-V7 region by Illumina sequencing platform

| Samples | Raw_Tags | Clean_Tags | Final_tags | OTUs |
| --- | --- | --- | --- | --- |
| nD | 34471±4134 | 33702±4157 | 31481±3907 | 164±10 |
| nS | 39970±1548 | 38181±2064 | 34922±2538 | 358±209 |
| SDY | 46272±5104 | 37475±4260 | 20591±1818 | 1528±39 |
| Ss | 83145±44123 | 70442±40229 | 43727±23594 | 1797±246 |

Table S2 the alpha diversity index of 16S V5-V7 region replicon sequencing of two root zone and two nodules of wild soybean grown in saline-alkali soil.

| Samples | chao1 | goods_coverage | observed_species | PD_whole_tree | shannon |
| --- | --- | --- | --- | --- | --- |
| nD | 356.94±10.90 | 0.9952±0.0005 | 104±15.8 | 30.8±10.5 | 0.15±0.04 |
| nS | 548.26±293.77 | 0.9910±0.0053 | 233.2±152.5 | 65.5±33.1 | 0.39±0.25 |
| SDY | 1737.90±14.38 | 0.9794±0.0005 | 1466.3±12.6 | 118.4±2.7 | 9.00±0.04 |
| Ss | 1867.28±99.50 | 0.9764±0.0006 | 1540.8±101.1 | 157.7±18.7 | 8.95±0.19 |

Table S3 The relative abundance of 16S rRNA in root zone of wild soybean

| Phylum | Relative abundance in root zone in coastal region（%） | Relative abundance in root zone in the Yellow river delta（%） |
| --- | --- | --- |
| Proteobacteria | 39.67±2.87 | 39.67±3.09 |
| Actinobacteria | 26.00±2.94 | 24.00±3.74 |
| Bacteroidetes | 10.67±1.70 | 9.00±0.82 |
| Chloroflexi | 6.00±0.05 | 6.67±0.47 |
| Acidobacteria | 3.67±0.90 | 6.00±0.02 |
| Gemmatimonadetes | 2.67±0.47 | 4.67±0.47 |
| Planctomycetes | 3.00±0.83 | 3.67±0.47 |
| Saccharibacteria | 1.33±0.47 | 0.20±0.00 |
| Firmicutes | 1.30±0.50 | 1.00±0.01 |
| Armatimonadetes | 0.67±0.05 | 0.73±0.09 |
| TM6 | 0.31±0.20 | 0.06±0.02 |
| Chlamydiae | 0.40±0.22 | 0.16±0.06 |
| Nitrospira | 0.67±0.21 | 0.67±0.12 |
| Verrucomicrobia | 0.90±0.14 | 2.00±0.00 |
| Parcubacteria | 0.50±0.37 | 0.04±0.01 |
| Cyanobacteria | 0.09±0.02 | 0.04±0.02 |

Table S4 The relative abundance in the nodules of wild soybean at phylum level

| Phylum name | nodule in coastal sand (nS) | nodule in the Yellow River delta soil (nD) |
| --- | --- | --- |
| Proteobacteria | 98.458±0.045 | 99.703±0.002 |
| γ-proteobacteria | 0.225±0.081 | 0.178±0.062 |
| β-proteobacteria | 0.187±0.052 | 0.053±0.010 |
| δ-proteobacteria | 0.134±0.061 | 0.046±0.010 |
| Actinobacteria | 0.533±0.340 | 0.167±0.047 |
| Bacteroidetes | 0.177±0.158 | 0.037±0.017 |
| Chloroflexs | 0.123±0.122 | 0.047±0.009 |
| Acidobacteria | 0.113±0.092 | 0.043±0.012 |
| Gemmatimonadetes | 0.117±0.095 | 0.043±0.009 |
| Planctomycetes | 0.077±0.057 | 0.014±0.008 |
| Saccharibacteria | 0.026±0.023 | 0.001±0.001 |
| Firmicutes | 0.036±0.031 | 0.010±0.007 |
| Armatimonadetes | 0.014±0.012 | 0.005±0.002 |
| TM6 | 0.010±0.009 | ND |
| Chlamydiae | 0.009±0.007 | ND |
| Nitrospira | 0.013±0.010 | ND |
| Verrucomicrobia | 0.022±0.016 | 0.004±0.001 |

ND: not detected

Table S5 The relative abundance of 16S rRNA in the nodules at genus level

| Genus | nS (%) | nD (%) |
| --- | --- | --- |
| Actinobacteria |  |  |
| *Microbacterium* | 0.066±0.009 | 0.006±0.002 |
| *Arthrobacter* | 0.060±0.010 | 0.008±0.004 |
| *Nocardiodes* | 0.059±0.011 | 0.009±0.002 |
| *Streptomyces* | 0.037±0.012 | 0.006±0.003 |
| *Blastococcus* | 0.012±0.003 | 0.017±0.005 |
| *Patulibacter* | 0.013±0.007 | 0.008±0.001 |
| Bacteroidetes |  |  |
| *Flavobacterium* | 0.042±0.010 | 0.001±0.001 |
| *Flavisolibacter* | 0.028±0.008 | 0.009±0.002 |
| Proteobacteria |  |  |
| *Sinorhizobium/Ensifer* (α-) | 97.330±1.701 | 98.661±0.471 |
| *Sphingomonas* (α-) | 0.086±0.009 | 0.016±0.004 |
| *Varribacter* (α-) | 0.015±0.006 | 0.013±0.004 |
| *Devosia* (α-) | 0.008±0.001 | 0.002±0.001 |
| *Pedomicrobium* (α-) | 0.008±0.001 | 0.014±0.005 |
| *Microvirga* (α-) | 0.006±0.001 | 0.008±0.002 |
| Enterobacteriaceae (γ-) | 0.086±0.005 | 0.056±0.009 |
| *Pseudomonas* (γ-) | 0.032±0.008 | 0.027±0.007 |
| *Acinetobacter* (γ-) | 0.012±0.003 | 0.002±0.000 |
| *Pseudoxanthomonas* (γ-) | 0.010±0.003 | 0.014±0.004 |
| *Candidatus- Entotheonella* (δ-) | 0.015±0.002 | 0.009±0.002 |
| *GR-WP33-30* (δ-) | 0.016±0.005 | 0.008±0.003 |
| *Methylibium* (β-) | 0.015±0.004 | 0.001±0.000 |
| *Ramlibacter* (β-) | 0.004±0.003 | 0.007±0.003 |
| Nitrosomonadaceae (β-) | 0.025±0.009 | 0.010±0.006 |
| Firmicutes |  |  |
| *Bacillus* | 0.022±0.005 | 0.010±0.007 |
| *Paenibacillus* | 0.008±0.003 | ND |
| *Halobacillus* | 0.004±0.002 | ND |
| *Lysinibacillus* | 0.004±0.001 | ND |

Table S6 Sequencing data of *nifH* gene fragments by Illumina platform

| Samples | Raw_Tags | Clean_Tags | Final_tags | OTUs |
| --- | --- | --- | --- | --- |
| nS | 67618±4707 | 67017±4505 | 66460±4358 | 194±36 |
| nD | 69471±6228 | 68701±6364 | 68083±6225 | 303±16 |
| Ss | 134675±28132 | 131744±27556 | 123471±26427 | 1689±21 |
| SDY | 150932±23230 | 148306±22497 | 136043±20027 | 2795±415 |

Table S7 the alpha diversity index of *nifH* replicon sequencing of two root zone and two nodules of wild soybean grown in saline-alkali soil.

| Samples | chao1 | goods_coverage | observed_species | PD_whole_tree | shannon |
| --- | --- | --- | --- | --- | --- |
| nD | 546.78±72.07 | 0.9973±0.0003 | 269.1±17.5 | 141.9±11.8 | 0.75±0.20 |
| nS | 341.96±39.64 | 0.9983±0.0002 | 174.1±29.3 | 107.3±17.4 | 0.61±0.32 |
| SDY | 2783.83±364.99 | 0.9887±0.0019 | 1334.7±303.4 | 953.3±191.5 | 7.61±0.28 |
| Ss | 1796.96±101.75 | 0.9922±0.0007 | 1327.0±114.1 | 561.6±98.7 | 6.54±0.45 |

Table S8 the relative abundance of *nifH* in nodules at phylum level

| type | Relative abundance in nS | Relative abundance in nD |
| --- | --- | --- |
| *Sinorhizobium/Ensifer* | 96.657±0.943 | 93.342±2.645 |
| Proteobacteria | 97.010±0.816 | 94.001±2.944 |
| Unidentified | 3.029±0.735 | 5.675±2.625 |
| Viridiplantae | ND | 0.005±0.003 |
| Euryarchaeota | ND | 0.004±0.003 |

Table S9 the relative abundance of *nifH* in root zones at phylum level

| type | SS | SDY |
| --- | --- | --- |
| Cyanobacteria | 49.000±20.607 | 37.667±8.055 |
| Proteobacteria | 32.323±9.843 | 31.333±3.091 |
| Unidentified | 5.672±0.473 | 10.333±2.357 |
| Delta/epsilon | 10.000±7.204 | 3.667±0.471 |
| Firmicutes | 0.668±0.231 | 4.104±0.393 |
| Actinobacteria | 0.238±0.047 | 9.000±2.160 |
| Euryarchaeota | 0.100±0.007 | 1.733±1.159 |
| Viridiplantae | 0.003±0.002 | 0.003±0.000 |
| Metazoa | 0.003±0.001 | 0.011±0.010 |
| Dickarya | 0.005±0.003 | 0.007±0.001 |

Table S10 the relative abundance of *nifH* in root zones at genus level

| Genus name | Relative abundance of niH gene in each phylum at samples Ss (%) | Relative abundance of niH gene in each phylum at samples SDY (%) |
| --- | --- | --- |
| Cyanobacteria | 100 | 100 |
| *Nostoc* | 58.7±9.9 | 40.3±0.5 |
| *Anabaena* | 13.7±1.7 | 40.0±1.6 |
| *Cylindrospermum* | 6.0±3.9 | 1.8±1.6 |
| *Gloeothece* | 5.5±3.5 | ND |
| *Cyanothece* | 12.0±8.5 | ND |
|  |  |  |
| Proteobacteria | 100 | 100 |
| *Bradyrhizobium* | 32.7±4.7 | 8.3±0.5 |
| *Agrobacterium_* | 1.7±0.5 | 10.3±7.9 |
| *Sinorhizobium* | 4.0±0.0 | 3.7±1.7 |
| *Azohydromonas* | 6.0±1.4 | 3.3±0.5 |
| *Paraburkholderia* | 5.0±2.8 | 4.0±3.5 |
| *Azospirillum* | 4.3±1.9 | 5.7±0.5 |
| *Azotobacter* | 1.7±0.5 | 1.0±0.5 |
| *Skermanella* | 4.3±0.5 | 9.7±2.0 |
| *Agrobacterium* | 1.7±0.5 | 10.3±4.8 |
| *Ectothiorhodospira* | 2.3±1.0 | 0.7±0.3 |
| *Methylobacter* | 0.4±0.1 | 17.7±6.2 |
|  |  |  |
| Actinobacteria | 100 | 100 |
| *Frankia* | 80.0±11.2 | 98.7±0.5 |
|  |  |  |
| Firmicutes | 100 | 100 |
| *Cellulosilyticum* | 35.3±33.0 | 38.3±4.5 |
| *Paenibacillus* | 24.0±12.3 | 37.3±6.0 |
| *Clostridium* | 13.3±7.8 | 14.3±6.2 |
| *Bacillus* | 12.7±7.0 | 1.4±0.8 |
